# Institutional Policy Changes Aimed at Addressing Obesity Among Mental Health Clients

**Published:** 2010-04-15

**Authors:** Linda L. Knol, Kelly Pritchett, Jeri Dunkin

**Affiliations:** University of Alabama; Central Washington University, Ellensburg, Washington; University of Alabama, Tuscaloosa, Alabama

## Abstract

**Background:**

People with mental illness often experience unique barriers to healthy eating and physical activity. For these clients, interventions should focus on changes in the immediate environment to change behaviors. The purpose of this project was to implement and evaluate policy changes that would limit calorie intake and increase calorie expenditure of clients receiving mental health services.

**Context:**

This intervention was implemented in a rural mental health system in the southeastern United States. Clients live in small group homes, where they are served breakfast, dinner, and a snack, and attend outpatient day treatment programs, where they are served lunch and can purchase snacks from vending machines.

**Methods:**

This intervention included institutional policy changes that altered menus and vending machine options and implemented group walking programs. Primary outcome measures were changes in clients' weight at 3 and 6 months after policy implementation.

**Consequences:**

At the 3-month follow-up, the median weight loss for overweight/obese clients (n = 45) was 1.4 kg. The 33 overweight/obese clients who were still in the group homes at the 6-month follow-up either maintained or continued to lose weight.

**Interpretation:**

Institutional policy changes aimed at improving dietary intake and physical activity levels among clients receiving mental health services can promote weight loss in overweight clients.

## Background

People with mental illness have reduced life expectancies and higher rates of cardiovascular disease than the general population (1-3). Approximately two-thirds of people with a mental illness have at least 1 comorbid health condition related to lifestyle choices (4,5). Compared with the general population, people with mental illness are more likely to have a sedentary lifestyle (3,6) and eat an unhealthful diet (3,6,7). It is difficult to determine whether people with mental illness are more likely to be overweight or obese than the general public on the basis of the current literature (8). However, the prevalence of overweight and obesity is high in this population and should be addressed.

People with mental illness encounter unique barriers to healthy eating and regular physical activity. Limited self-management skills or knowledge deficits lead to relying on readily available convenience foods and low levels of planned exercise. Other barriers that affect healthy eating and physical activity include poverty, unemployment, limited transportation, and financial exploitation by others (3,6,9). In addition to these barriers, people with mental illness experience side effects to medications that prevent weight loss or promote weight gain such as increased appetite, food cravings, and lethargy.

In 2005, the US Surgeon General advocated for services that promote wellness among people with mental illness (10). A logical point of access in this population is through mental health services. Therefore, wellness programs and policies should be integrated into the system of mental health services (11). Foods served or sold in mental health institutions should meet standards such as the 2005 Dietary Guidelines for Americans (12). Mental health institutions should adopt policies that support opportunities to engage in physical activity, such as outdoor activities, active field trips, exercise classes, and gardening (13).

A review of behavioral management of weight gain in psychiatric patients concluded that successful programs addressed weight management through individual counseling on lifestyle choices and caloric restriction in a controlled environment, such as inpatient hospital care (14). Results of these studies were not always significant because of small sample sizes and short intervention periods and may not apply to community-dwelling clients. To date, no program has investigated whether the adoption of wellness policies by a mental health system would decrease weight of community-dwelling people with mental illness. The purpose of this intervention was to reduce weight in overweight, community-dwelling clients with mental illness who live in a rural area.

## Context

This intervention was implemented in a mental health system in a rural area in the southern United States. This system covers 5 counties, or a 8,800 km^2^ region, and includes 300 residential beds for the mentally ill. Community-dwelling clients are housed in either transitional group homes or apartments. This intervention was pilot tested in 5 transitional group homes for clients with mental illness in 3 of the 5 counties. The number of beds in each group home varied (range, 10-50). People seeking mental health services may cycle through periods of stability and instability. As their symptoms improve and they gain independence, they may move from a transitional group home to an apartment or house with relatives or friends. The length of time that a client may stay in transitional group housing varies according to the client's mental status and the complexity of treatment for comorbid conditions.

The group homes serve breakfast, dinner, and an evening snack. Each group home employs its own cook. Most of the cooks were not familiar with equipment used to portion foods in commercial food service operations. At the start of the program, the largest group home had already adopted a series of planned menus that repeated in a 4-week cycle, while the 4 smaller group homes allowed residents to create their own menus with supervision. Residents were allowed to eat second helpings of all items served at meals. The largest group home was situated on a large piece of land with outdoor space for clients to walk, and the 4 smaller group homes were situated on small plots of land just off curvy, rural roads without sidewalks. Few outdoor activities were planned, but clients at each group home had opportunities to participate daily in light calisthenics led by an activity director.

Most clients attend day treatment programs on weekdays, coordinated by the mental health system. These programs are housed in a building that is centrally located in each county. Clients take a shuttle bus to the closest day treatment program. While attending day treatment, clients are served lunch and can buy snacks through vending machines or the canteen. Before implementing any policy changes, we audited vending machines in the day treatment buildings. Items varied in calorie and fat content. Only 4 of 40 snack items sold in vending machines in these facilities had fewer than 150 kcal and 5 g total fat per serving. Only 4 of 12 beverage options sold in the vending machines were "diet" or contained fewer than 10 kcal. The options sold in the canteen were slightly less caloric than those sold in vending machines.

## Methods

### Intervention

From fall 2007 to fall 2008, we planned and implemented several environmental changes that addressed calorie intake and expenditure. Administrators, key staff members, and clients provided guidance on how to best implement the program. Policy changes were implemented to improve or maintain weight status and directed toward 3 potential behavioral outcomes: reducing portion sizes at meals, reducing calorie content of snacks to less than 200 kcal, and increasing moderate physical activity to at least 30 to 60 minutes daily.

For all menus, a 1,600-kcal/day meal pattern was established on the basis of 3 meals and 3 small snacks. Meals were planned to meet the 2005 Dietary Guidelines for Americans and current evidence-based guidelines for weight management (12,15). Because of the large percentage of clients with diabetes, meals and snacks were planned to contribute consistent amounts of carbohydrate. The estimated calorie requirement for sedentary women is approximately 1,800 kcal/day and for men is approximately 2,200 kcal/day (12). Therefore, the caloric level of the menus seemed appropriate for slow, gradual weight loss in overweight clients and weight maintenance in normal-weight clients. In an effort to retrain clients to eat smaller portions, second helpings at meals were limited to low-fat, low-carbohydrate menu items, such as steamed vegetables and salads. The beverages served at all facilities were changed to sugar-free, caffeine-free beverages or 1% or skim milk. In addition, only packets of nonnutritive sweeteners were available in dining rooms. Nursing staff monitored clients' weight. Clients whose weight fell in the underweight or normal-weight range and who were having difficulty maintaining weight received additional food at meals. Staff members and food service employees received educational in-services on menu changes and proper portion control. Clients were involved in developing menus so that favorite items would continue to appear on the menus. We worked with the food service staff to solve problems related to acquisition, preparation, and client acceptance of new menu items.

Our rural clients have limited access to grocery and convenience stores, so more of their income may be used to purchase items from vending machines and canteens in the day treatment facilities. After the vending machine audit, the board of directors adopted a policy change that limited vending machine options to sugar-free, caffeine-free beverages and single-item snacks that contain fewer than 150 kcal, 5 g total fat, and 15 g carbohydrate. Before the policy change, a snack purchased in a vending machine could contain up to 100 g carbohydrate and 700 kcal. The day treatment facilities manage and stock the vending machines and canteens, so changing options in the machines was easy. A variety of prepackaged 100-kcal snack items were purchased from local grocery stores and stocked in the vending machines.

We developed a group walking program for the clients based on guidelines from the 2005 Dietary Guidelines for Americans (12), but only 1 group home was able to implement this walking program because of location. Duration and intensity of walking were based on each client's ability. Staff members encouraged clients to walk at the same time each day, which corresponded with a lull in planned activities. Clients at the group home where the walking program was implemented enjoyed the opportunity to walk outdoors on days when the weather was nice.

Educational components of the program focused on the rationale for the policy changes. Most clients understood that the changes had been made to improve their health. In the future, we plan to incorporate educational components into existing basic living skills courses, with the help of course instructors.

### Outcome evaluation

After the policy changes were implemented, a retrospective chart review was completed to determine effectiveness of the program. Before data were collected, all procedures were approved by the institutional review board of the University of Alabama. We asked clients to complete an informed consent and disclosure waiver that allowed researchers to access their medical records to obtain demographic information and medical and psychiatric diagnoses. We report data only for clients who signed the informed consent and disclosure waivers. One of the biggest challenges of the project was the evaluation. Every effort was made to obtain consent from the clients who lived in or transitioned through the group homes that implemented the policy changes. However, some clients transitioned through group home living quickly, and we missed the opportunity to invite them to participate in the evaluation. Other clients refused to participate or could not provide consent. Another challenge for the evaluation team was loss to follow-up. The changes we report in weight represent only clients who stayed in the group homes for 3 and 6 months, which was a small percentage of the original cohort.

We obtained height and weight through logs kept at each group home. Each resident was weighed once per month. Height was measured once. Only data from clients who had both their height and weight measured were used in this evaluation (n = 65). We calculated body mass index and categorized participants into 1 of 4 groups: underweight (<18.5 kg/m^2^), normal weight (18.5-24.9 kg/m^2^), overweight (25.0-29.9 kg/m^2^), or obese (≥30.0 kg/m^2^) (16). We also rated adherence to policy changes in each of the 5 group homes. A level 1 site was defined as a site that complied with menu changes, had no sugar-sweetened beverages in vending machines, limited snack items in vending machines and canteens to only those recommended, and implemented a walking program. Only 1 group home fit this description, but it housed the most clients (50 beds). A level 2 site was defined as a site that complied with some but not all 4 of the policy changes. The other 4 group homes fell into this category because they were able to make all the policy changes except implementing an outdoor walking program. These homes housed up to 15 people and were on smaller lots with no available sidewalks.

## Consequences

Among the 121 clients who lived in transitional group homes after the policy changes were implemented, 73 (60%) signed an informed consent form and disclosure waiver that allowed their health information to be used to evaluate the policy changes. Height and weight data were available for 65 of these clients (Table 1). The average age of the group was 50 (standard deviation, 12; range, 19-76) years.

Three months after the policy was implemented, 26 (58%) of the 45 overweight/obese residents had lost weight (range, 0.5-10.9 kg). After 6 months, 17 (52%) of the 33 overweight/obese residents still living in a group home had lost weight (range, 0.7-18.6 kg). In the home that fully implemented all policy changes, 11 (69%) of 16 overweight/obese residents had lost weight at 3 months, and 8 (67%) of 12 overweight/obese residents had lost weight at 6 months. By comparison, 15 (52%) of 29 and 9 (43%) of 21 overweight/obese residents of group homes where the policy changes were partially implemented lost weight during the 3- and 6-month follow-up periods, respectively.

Normal-weight clients gained a median of 2.2 kg at 3 months and 1.8 kg at 6 months (Table 2). Two of the 11 normal-weight clients lost weight during this 6-month period but remained in the normal-weight range.

## Interpretation

Preliminary results from this intervention suggest that small institutional policy changes promoted weight loss in slightly more than half of overweight clients without producing weight loss in normal-weight clients. The weight loss was sustained for 6 months in 52% of the overweight clients. Of note, these clients were not trying to lose weight or required to participate in the walking program.

A novel approach to this intervention was the focus on institutional policy change. Most weight control programs for clients with mental illness have focused on overweight clients or those at risk of gaining weight because of medication use. This program focused on healthy eating and physical activity for all clients. The administration and staff fully supported the policy changes. Without their support and input into the program, the policy changes could not have been implemented. In this instance, we used consensus among administration, front line employees, and clients to solve problems associated with policy changes.

Although we had administrative support, we encountered barriers. Depending on the cooking skills and nutrition knowledge of food service personnel, menu changes were easier to implement at some group homes than others. Many of the group homes purchased food in bulk from local discount grocery stores. Obtaining foods such as whole wheat rolls, fresh fruit, or lean meats was difficult. The primary component of the physical activity program was outdoor walking. This activity was selected because of budget constraints and ease of implementation. During temperate months, approximately 25% of the clients in the largest group home walked daily; however, participation decreased during unusually hot or cold months. For some clients, the walking program was an opportunity for access to the outdoors, which was otherwise limited. Unfortunately, the 4 smaller group homes could not implement the walking program because of safety concerns related to their location.

The intent of this project was to evaluate the efficacy of implementing a few policy changes to improve the food and physical activity environment of mental health clients. Unfortunately, the brief length of stay in transitional group housing for most of these clients impaired our ability to obtain informed consent for the retrospective evaluation of weight changes. Additionally, some clients could not provide consent because of altered mental status. Therefore, we cannot document the weight changes for all clients affected by the policy change, which may confound the results. As with any policy research, we cannot verify that all policies were followed on all days of the week at all the intervention sites. Furthermore, residents were free to buy snacks they could keep in their rooms and dine out in restaurants with friends and family. Although vending options were changed, client purchases were not limited. This study did not assess dietary or physical activity changes in the sample or other factors that may confound the relationship between policy changes and clients' weight. Future research should collect data on these factors.

In conclusion, when supported by the management, these policy changes were easy to implement, produced some weight loss in the desired population, and may be sustainable long-term.

## Figures and Tables

**Table 1:** Characteristics of Clients With Mental Illness Living in 5 Rural Group Homes, Southeastern United States

**Characteristic**	All Group Homes (n = 65), n (%)^a^	Level 1 Group Home^b^ (n = 25), n (%)^a^	Level 2 Group Homes^b^ (n = 40), n (%)^a^
**Sex**			
Female	29 (45)	11 (44)	18 (45)
Male	36 (55)	14 (56)	22 (55)
**Race^c^**			
White	46 (73)	17 (71)	29 (74)
African American	16 (25)	6 (25)	10 (26)
Other	1 (2)	1 (4)	0
**Weight**			
Underweight	1 (2)	0	1 (2)
Normal weight	14 (22)	5 (20)	9 (22)
Overweight	18 (28)	10 (40)	8 (20)
Obese	32 (49)	10 (40)	22 (55)
**Comorbid conditions^c^**			
Hypertension	32 (53)	15 (60)	17 (49)
Diabetes	23 (38)	10 (40)	13 (37)
Hyperlipidemia	22 (37)	11 (44)	11 (31)

a Percentages may not total 100 because of rounding.

b Level refers to the degree to which the environmental components of the intervention were carried out. A level 1 site complied with menu changes, had no sugar-sweetened beverages in vending machines, limited snack items, and started and maintained a walking program. Four level 2 sites met all criteria listed above but did not start a walking program.

c Data for these characteristics are missing for some participants. Percentages were calculated on the basis of available data.

**Table 2:** Weight Change by Level of Intervention^a^ and Initial Weight Status in a Sample of Clients With Mental Illness Living in 5 Rural Group Homes, Southeastern United States

Initial Weight Status	Baseline		3 Months		6 Months	

	No. of Clients	Mean (SD) Weight, kg^b^	No. of Clients	Change (IQR), kg^c^	No. of Clients	Change (IQR), kg^d^
**Both levels**						
Normal weight	15	64.8 (8.3)	13	2.2 (−0.5 to 2.7)	11	1.8 (0.0 to 6.4)
Overweight/obese	50	93.7 (18.9)	45	−1.4 (−2.3 to 1.4)	33	−0.7 (−2.7 to 3.2)
**Level 1**						
Normal weight	5	63.1 (6.2)	4	0.8 (−1.5 to 1.8)	2	4.1 (1.8 to 6.4)
Overweight/obese	20	88.1 (20.3)	16	−1.8 (−3.0 to 0.9)	12	−1.4 (−3.2 to 0.7)
**Level 2**						
Normal weight	10	65.7 (9.4)	9	2.3 (−0.5 to 2.7)	9	1.6 (0.0 to 5.0)
Overweight/obese	30	97.5 (17.2)	29	−0.5 (−1.8 to 1.8)	21	0.5 (−2.7 to 3.2)

Abbreviations: SD, standard deviation; IQR, interquartile range.

a Level refers to the degree to which the environmental components of the intervention were carried out. A level 1 site complied with menu changes, had no sugar-sweetened beverages in vending machines, limited snack items, and started and maintained a walking program. Four level 2 sites met all criteria listed above but did not start a walking program.

b Average weight of clients living in transitional group homes before the policy changes or on entry into transitional housing.

c Median change in weight from baseline for clients living in transitional group homes for at least 3 months after the initial policy changes.

d Median change in weight from baseline for clients living in transitional group homes for at least 6 months after the initial policy changes.
